# A novel frameshift mutation of *SMPX* causes a rare form of X-linked nonsyndromic hearing loss in a Chinese family

**DOI:** 10.1371/journal.pone.0178384

**Published:** 2017-05-25

**Authors:** Zhijie Niu, Yong Feng, Lingyun Mei, Jie Sun, Xueping Wang, Juncheng Wang, Zhengmao Hu, Yunpeng Dong, Hongsheng Chen, Chufeng He, Yalan Liu, Xinzhang Cai, Xuezhong Liu, Lu Jiang

**Affiliations:** 1 Department of Otolaryngology-Head and Neck Surgery, Xiangya Hospital, Central South University, Changsha, PR China; 2 Key Laboratory of Otolaryngology Major Disease Research of Hunan Province, Changsha, PR China; 3 Department of Otolaryngology (D-48), University of Miami Miller School of Medicine, Miami, FL, United States of America; 4 State Key Laboratory of Medical Genetics, Central South University, Changsha, PR China; NIDCR/NIH, UNITED STATES

## Abstract

X-linked hearing impairment is the rarest form of genetic hearing loss (HL) and represents only a minor fraction of all cases. The aim of this study was to investigate the cause of X-linked inherited sensorineural HL in a four-generation Chinese family. A novel duplication variant (c.217dupA, p.Ile73Asnfs*5) in *SMPX* was identified by whole-exome sequencing. The frameshift mutation predicted to result in the premature truncation of the SMPX protein was co-segregated with the HL phenotype and was absent in 295 normal controls. Subpopulation screening of the coding exons and flanking introns of *SMPX* was further performed for 338 Chinese patients with nonsydromic HL by Sanger sequencing, and another two potential causative substitutions (c.238C>A and c.55A>G) in *SMPX* were identified in additional sporadic cases of congenital deafness. Collectively, this study is the first to report the role of *SMPX* in Chinese population and identify a novel frameshift mutation in *SMPX* that causes not only nonsyndromic late-onset progressive HL, but also congenital hearing impairment. Our findings extend the mutation and phenotypic spectrum of the *SMPX* gene.

## Introduction

Hearing loss (HL) is one the most common sensory diseases affecting approximately 1/1000 newborns and 360 million people worldwide (WHO data, http://www.who.int), and more than two-thirds of HL cases can be attributed to genetic causes. Hereditary HL is a genetically heterogeneous disorder [1], which is classified into syndromic hearing loss (SHL, 30%) and nonsyndromic hearing loss (NSHL, 70%). Up to now, 150 loci have been linked to NSHL, and over 100 genes have been identified (Van Camp G, Smith RJH. http://hereditaryhearingloss.org). The genes responsible for NSHL are involved in a wide variety of functions [2]. The reported mutations associated with NSHL are predominantly located in autosomes. X-linked nonsyndromic deafness is relatively rare and accounts for only 1%~5% of all cases and approximately 5% of cases of pre-lingual male deafness [3]. Phenotypic characteristics of males often include an earlier onset and more severe presentation than females due to X-inactivation [4]. Currently, only six known NSHL loci have been mapped to the X chromosome, including the following five DFNX genes: *PRPS1* (DFNX1, OMIM 304500), *POU3F4* (DFNX2, OMIM 304400), *SMPX* (DFNX4, OMIM 300066), *AIFM1* (DFNX5, OMIM 300614), *COL4A6* (DFNX6, OMIM 303630).

The human *SMPX* gene maps to chromosome X at p.22.1 and consists of five exons, with the first and the fifth exon being non-coding [5]. The gene is highly conserved across mammalian species [6]. *SMPX* encodes the small muscle protein with no known functional domains and is suggested to play an important role in inner-ear development and/or maintenance through interactions with various regulators such as insulin-like growth factor 1 (IGF-1) [6, 7], integrins (α8β1) and Rac1 [8, 9]. In the inner ear, Smpx is expressed in different cell types of the murine cochlea, including Böttcher cells, pillar cells, root cells, interdental cells of the limbus spiralis and in relatively low levels in hair cells [10]. With the development of high-throughput sequencing, whole-exome sequencing (WES) has been successfully employed to identify the pathogenic variations in Mendelian disorders [11, 12]. For many NSHL cases, more than 10 causative genes have recently been identified via WES. WES is now an effective alternative strategy for the identification of disease-causing variants in unexpected genes.

Here, we identified a novel frameshift duplication mutation (c.217dupA, p.Ile73Asnfs*5) of *SMPX* in a four-generation X-linked family from China by WES. To summarize the mutation spectrum of *SMPX* in a Chinese population, we then screened 338 nonsyndromic patients with various severities of HL using regular Sanger sequencing and identified another two variations (c.238C>A and c.55A>G), which are likely responsible for the phenotypes in these patients. To date, no DFNX4 cases were previously reported among Asians, and this is the first report of mutations associated with DFNX4 in Asian patients.

## Materials and methods

### Subjects and clinical evaluations

**Fig 1:**
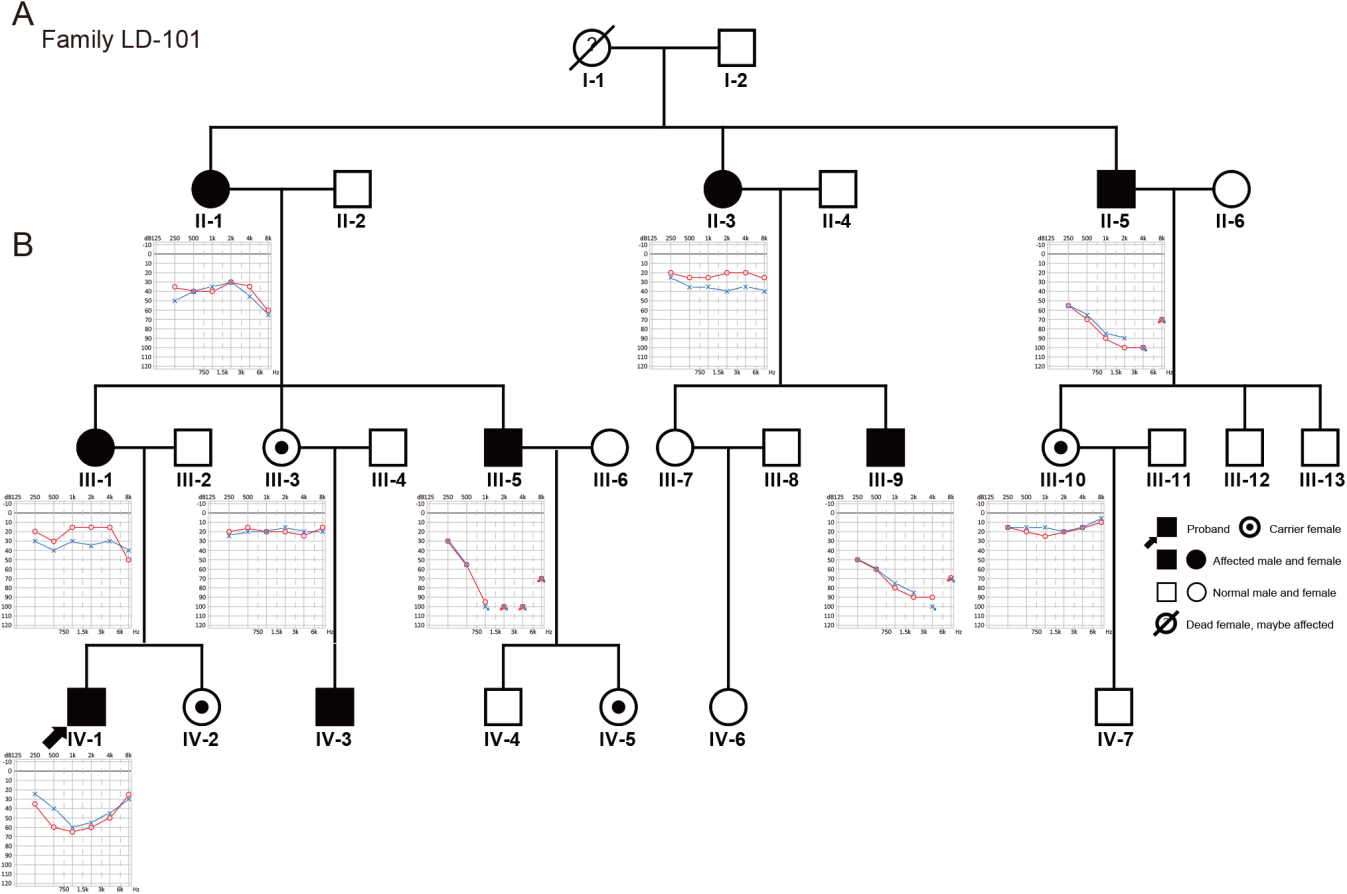
Pedigree and clinical phenotype. (A): Pedigree of family LD-101 with hearing loss. This pedigree demonstrates nonsyndromic hearing loss of X-linked inheritance. Open symbols, unaffected; solid symbols, affected. Squares, males; circles, females; slashed, deceased individual; and slanting arrow, the proband. (B): Audiograms of nine family members (red indicates the right ear, blue indicates the left ear). The age at onset of hearing loss in males varied from childhood to 8 years of age, whereas hearing loss in females began in the adulthood and varied among the individuals.

This four-generation family of Han origin (designated as LD-101) with NSHL was found in Hunan, China and the pedigree was constructed (Fig 1A). The pattern of inheritance suggested X-linked dominant inheritance. This study was approved by the Medical Ethics Committee of Xiangya Hospital, Central South University. Written informed consent was obtained from all of the study participants or, in case of children, from their parents. A detailed medical history of all participating family members was obtained and collected by a questionnaire designed to assess the medical and environmental factors that may cause hearing loss. Physical and otological examinations of the participants were independently performed by two experienced otologists to exclude systematic abnormalities, including the use of an electric auriscope to exclude tympanitis and perforation. Pure-tone audiometry (PTA) and immittance were performed as the standard protocol in this family. Auditory brainstem response (ABR), auditory steady state response (ASSR), distortion product otoacoustic emission (DPOAE) and vestibular testing were carried out on particular individuals. Syndromic features were given special attention. The proband (IV-1, Fig 1A) and his cousin (IV-3, Fig 1A) underwent high-resolution computed tomography (HRCT) and/or magnetic resonance imaging (MRI), ophthalmic examination, routine blood examination and a urine test. The list of the classification criteria for hearing impairment was as follows: normal (< 25 dB HL), mild/slight (26 to 40 dB HL), moderate (41 to 60 dB HL), severe (60 to 80 dB HL) and profound HL (>81 dB HL).

A total of 88 patients of 59 families with NSHL and 250 sporadic deafness subjects (1998~2014) were recruited for further analysis in the current study. All selected families exhibited typical in dominant HL, and audiological assessments of the affected subjects revealed bilateral, sensorineural hearing loss. A total of 250 sporadic cases with NSHL and their probands were provided by the Otology Clinic of Xiangya Hospital of Central South University. The age of the selected sporadic individuals varied from 1 to 62 years. Clinical questionnaires were obtained from all individuals or their parents. All of the participants underwent audiological assessments. The selected subjects fulfilled the following criteria: i) NSHL with mild to profound pre-lingual or post-lingual hearing loss; ii) no detectable etiology according to medical records; iii) *GJB2*, *GJB3* and *mtDNA* A1555G mutations, which are prevalent in the Chinese deaf population, were excluded by standard genetic screening; and iv) informed and written consent was obtained from all subjects or their legal guardians. An additional 295 unrelated subjects with normal hearing were recruited as matched control in this study. All individuals were of Han Chinese ethnicity and geographic ancestry.

### DNA extraction and exome sequencing

Whole-blood samples were obtained from all participants, and all genomic DNA samples were extracted from collected blood samples using the standard phenol/chloroform method. Genomic DNA from three affected individuals (IV-1, IV-3 and III-9, Fig 1A) from family LD-101 was subjected to WES performed at the Novogene Bioinformatics Institute, Beijing, China. In massive parallel sequencing, qualified genomic DNA was randomly broken into fragments with an average length 180–280 bp using a Covaris S220 sonicator. Then, the Agilent SureSelect Human All Exon V5 kit was used to enrich, hybridize, and capture the obtained fragments according to the manufacturer’s procedures. Each captured DNA library was sequenced on a Hiseq 4000 platform (Illumina) with the desired average sequencing depth. The raw image files were processed with the Illumina pipeline for base-calling using default parameters. The acquired valid sequencing data were matched to the human reference genome (UCSC hg19) using Burrows-Wheeler Aligner (BWA) software to obtain the original mapping result, which was then sorted and marked with SAMtools and Picard software, respectively. SAMtools and bcftools were then used to perform variant calling on the collected reads that aligned to exon regions and to identify variants that were filtered against the dbSNP138 database, 1000 Genome Project, and NHLBI Exome Sequencing Project (ESP) 6500 Project. Functional prediction was carried out using PolyPhen-2, SIFT, MutationTaster, LRT and MetaLR. The candidate variants were annotated in ANNOVAR (Annotate Variation) software.

### Sanger sequencing

Sanger sequencing was used to confirm all shared variants of the three affected members after filtering. Based on the genomic sequences (GRCH37/hg19) from the human genome, PCR primers flanking the candidate loci were designed with Premier 5.0 software and synthesized by Genscript, Nanjing, China. The PCR conditions were as follows: an initial step at 95°C for 5 min; 15 cycles at 94°C for 30 s for denaturation, 62°C for 1 min for annealing, and 72°C for 1 min for extension; another 24 cycles of 94°C for 30 s, 56°C for 1 min, and 72°C for 11 min to ensure complete extention; and final storage at 4°C. The PCR products were purified and then sequenced using an ABI 3730 Genetic Analyzer (Applied Biosystems, Foster City, CA, USA). The DNASTAR V7.1 software suite and Chromas V2.4 software were used to analyze the sequences. Mutation analysis of the *SMPX* gene (NM_014332.1, hg19 X:21761891) in the Chinese deaf population was performed using the same method as described above. Identified causative nucleotide changes were named using Human Genome Variation Society (HGVS) nomenclature and submitted to MutationTaster for functional prediction.

## Results

### Clinical features

The affected subjects from family LD-101 showed an X-linked nonsyndromic sensorineural hearing impairment, which was more severe in males than in females (Fig 1B). PTA data revealed that the age at onset of disease was 0–8 years in males. The majority of affected males initially presented pre-lingual, bilateral, symmetric, progressive HL at high or middle frequencies, which then developed into severe to profound HL before their second decade of life. The newborn IV-3 failed the newborn hearing screening testing (NHST) and exhibited a bilateral, moderate-to-severe congenital hearing impairment in ABR testing, which was not been noticed in any other reported families. Even though the III-9, II-5 and III-5 subjects passed the NHST, they suffered from mild functional difficulty in speech recognition, suggesting that they may have had non-detectable mild HL when screened. Thereafter, the middle to high frequencies continued to deteriorate in the later years of the first decade and resulted in a profound loss of hearing. Therefore, these individuals could not be excluded from having congenital deafness. In contrast, females showed a variable age of onset and clinical severity. Subjects II-3 and III-1 both exhibited unilateral mild HL at the third and fourth decades of life, respectively. Subjects III-3 and III-10 who could well develop HL in the coming years did not present any signs of hearing impairment at last visit to our department. The detailed clinical features of these subjects are tabulated in Table 1.

**Table 1:** Summary of the clinical data of all participants from family LD-101.

Subjects	Gender	Age at test (years)	Age at onset (years)	Noise exposure	PTA^a^ (dB) Left ear	PTA^a^ (dB) Right ear	Audiogram shape	Tinnitus	Vertigo	Severity
**II-1**	Female	51	49	No	37.5	36.25	Flat	yes	No	mild
**II-3**	Female	49	—	No	36.25	22.5	—	No	No	normal
**II-4**	Male	48	—	No	28.75	23.75	—	No	No	normal
**II-5**	Male	45	child	No	85	90	Sloping	No	No	profound
**II-6**	Female	44	—	No	21.25	22.5	—	No	No	normal
**III-1**	Female	31	—	No	33.75	18.75	—	No	No	normal
**III-2**	Male	31	—	No	11.25	18.75	—	No	No	normal
**III-3**	Female	29	—	No	18.75	20	—	No	No	normal
**III-4**	Male	29	—	No	18.75	20	—	No	No	normal
**III-5**	Male	27	7~8	No	88.75	87.5	Sloping	yes	No	profound
**III-6**	Female	24	—	No	10	7.5	—	No	No	normal
**III-9**	Male	22	child	No	80	80	Sloping	No	No	normal
**III-10**	Female	24	—	No	16.25	20	—	No	No	normal
**III-12**	Male	20	—	No	8.75	11.25	—	No	No	normal
**III-13**	Male	18	—	No	11.25	13.75	—	No	No	normal
IV-1*	Male	7	5	No	50	58.75	U-shape	yes	No	moderate
**IV-3**	Male	4/12	0	No	No	No	—	—	—	severe

^a^ PTA = pure-tone average

*, proband

For all affected family members, tinnitus was not reported, except in three individuals (II-1, III-5, and IV-1, Fig 1A) who rejected noise exposure. There was no evidence for non-genetic causes of hearing impairment in this pedigree. No signs of a conductive HL component were noted in PTA and the acoustic immitance measurements, which indicated normal middle-ear function. There was also no any apparent evidence for vestibular dysfunction by vestibular testing. All ABR results of the three affected subjects (II-1, IV-3, and IV-1, Fig 1A) revealed cochlear involvement in accordance with the PTA and DPOAE of these patients. The HRCT and MRI results showed no signs of malformation in the proband and his cousin (IV-1 and IV-3, Fig 1A). Furthermore, there were no abnormal results from the ophthalmic examination or blood and urine tests. Comprehensive examination of the individuals of this family did not demonstrate any other clinical syndromic associated features.

### Analysis of the WES data

WES were performed for three affected family members (S1 Table) to identify the disease-causing gene. We obtained an average of 6.57 Gb of raw data, with at least 75X average coverage for each individual as paired-end reads. After mapping according to the human reference genome, ~99.9% of the targeted bases were covered sufficiently, and ~98.8% of the targeted region was covered at least 10X. The shared variants were filtered against dbSNP 138, the 1000 Genome Project, and NHLBI-ESP 6500 Project. The functional effects of non-synonymous SNPs were predicted using PolyPhen-2, SIFT, MutationTaster, LRT and MetaLR. Finally, 6 indels, which were located on the X chromosome, were considered to be the potential causative variants for NSHL in view of the X-linked inheritance of this family. The results for these three family members were confirmed by PCR sequencing.

### Co-segregation analysis of *SMPX* c.217dupA in LD-101 family members

**Fig 2:**
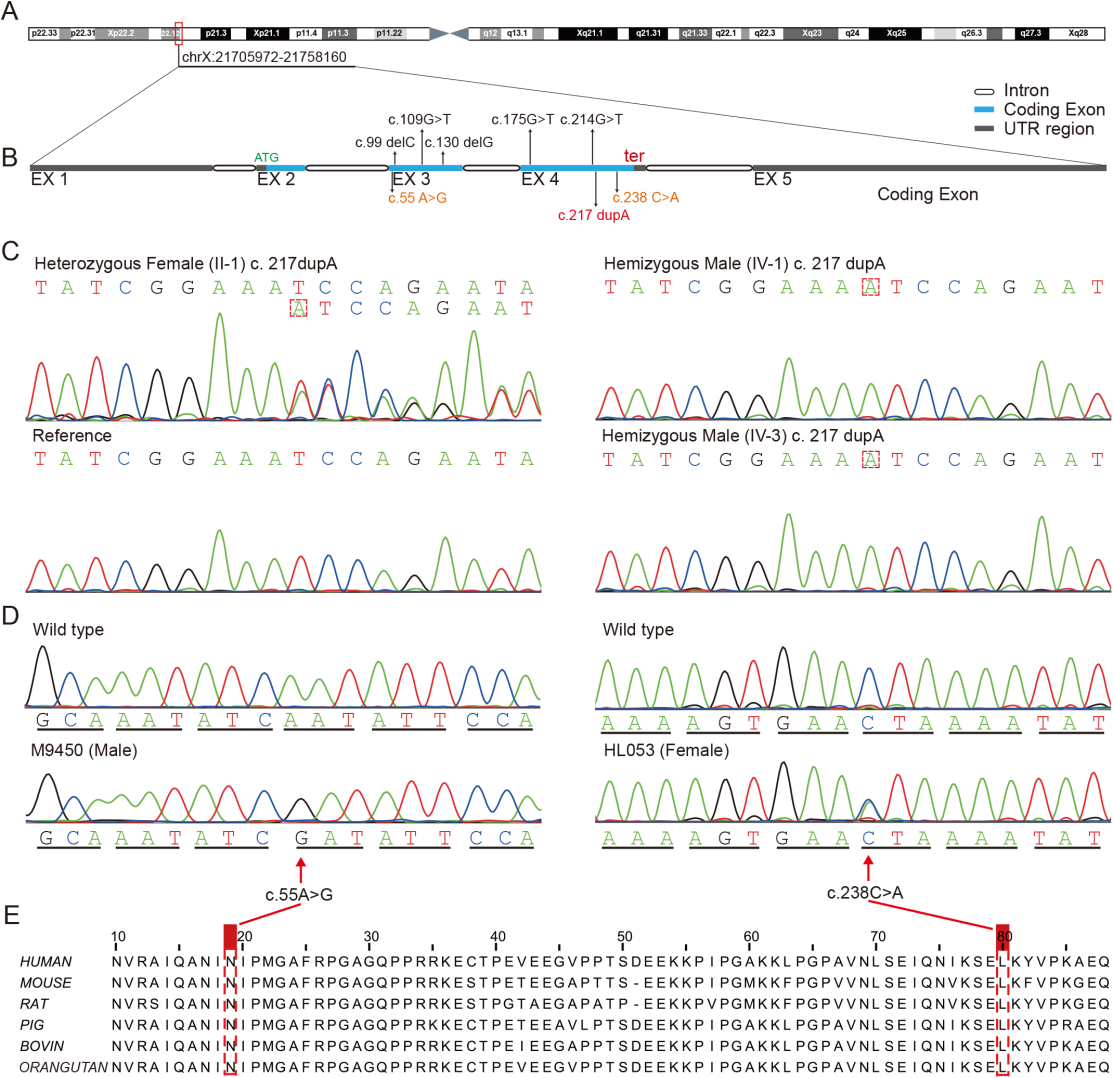
Schematic physical and genetic map of the *SMPX* gene (NM_014332.2, NP_055147, OMIM: 300066) and three variations found in family LD-101 and two additional sporadic cases. (A): Schematic physical and genetic maps of *SMPX*, located in the Xp21.12 chromosomal region (red bar). (B): Structure of the *SMPX* gene (5 exons) showing five known mutations (above the bar, black) and the identified variations in this study (below the bar, orange and red). (C): Sequencing chromatograms of *SMPX* showing the missense mutation c. 217dupA in exon 4 in the Chinese family. Electropherograms of a respective heterozygous female carrier (II-1) and two hemizygous male (IV-1 and IV-3) are shown in comparison to a reference sequence. The duplicated nucleotide is indicated by a red box. (D): Two identified variations likely responsible for the hearing loss phenotype in two *GJB2*-negative individuals (HL053 and M9450). The mutated nucleotide is indicated by red arrows. (E): Conservation analysis shows that Asn19Asp and Leu80Ile are highly conserved across human (Q9UHP9), mouse (Q9DC77), rat (Q925F0), pig (Q0MUU2), bovine (Q3ZBD4) and orangutan (Q5RB90).

After analysis of the results from WES, we used Sanger sequencing to identify the six validated indels (Table 2) in 20 biological family members, 8 affected relatives and 12 unaffected subjects. As a result, only one variant, c.217dupA, was located in exon 4 of *SMPX* and fully co-segregated with the HL phenotype of this family (Fig 2A–2C). The de novo insertion corresponding to codon 73 was found to cause a frameshift mutation after the 72^nd^ residue (Glu), and if translated, this mutation would terminate translation at codon 77 (p.Ile73Asnfs*5). The results showed that 5 males were hemizygous and 7 females were heterozygous for this mutation. However, this disease-causing variant was not detected in other unaffected relatives and 295 ethnicity-matched controls with normal hearing. Thus, Sanger sequencing of the coding exons and flanking regions of *SMPX* in all family members revealed no additional mutations in this gene.

**Table 2:** Candidate variants shared by three affected individuals.

Gene	Chromosome	Position	Reference	Alter	Func	Condon	RefSeq
***NHS***	ChrX	17705850	CTT	CTTT	splicing	c.566-10->T	NM_198270.3
***SMPX***	ChrX	21755730	ATTT	ATTTT	exonic	c.217dupA	NM_014332.2
***DCAF8L2***	ChrX	27765399		-6GGAGGA	exonic	c.429_434del	NM_001136533.1
***TCEAL6***	ChrX	101395780	TGGG	TGGGG	exonic	c.521dupC	NM_001006938.2
***GRIA3***	ChrX	122336600	TGGG	TGGGG	exonic	c.384dupG	NM_001256743
***GPR112***	ChrX	135474444	AGATGAT	AGAT	exonic	c.7969_7971del	NM_153834

### Screening of *SMPX* mutations in sporadic subjects and NSHL pedigrees

We performed Sanger sequencing of the three coding exons 2/3/4 and exon-intron boundaries from the collected 88 probands of other unrelated NSHL pedigrees and 250 sporadic NSHL persons. Sequence data were analyzed, and primer sequences for PCR amplification are provided in S2 Table. In two sporadic patients with deafness and dumbness, we detected two single nucleotide variations, c.238C>A (HL053, female, p.Leu80Ile) and c.55A>G (M9450, male, p.Asn19Asp), in the *SMPX* gene (Fig 2D). These variations were not detected among the 295 recruited controls of Han origin. The former variation was not reported in the Exome Variant Server and not detected at this position in the dbSNP or Exome Aggregation Consortium (ExAC) databases. The later variation has been annotated as a rare variant in dbSNP (rs759552778) and ExAC databases. However, there were no available family members for co-segregation analysis. To assess the pathogenicity of these genetic alterations, we calculated a pathogenicity score by summing the SIFT, PolyPhen-2, and MutationTaster scores to predict a functionally deleterious outcome.

## Discussion

In the present study, we identified a novel truncating variant (c.217dupA, p.Ile73Asnfs*5) in exon 4 of the *SMPX* gene in a multiplex family from China using WES techniques. Subsequently, this frameshift mutation was confirmed by Sanger sequencing and was absent in the unaffected family members and normal controls, demonstrating complete co-segregation with the HL phenotype in this family.

Although rare, an X-linked pattern of inheritance was detected in the present study based on the absence of male-to-male transmission, early disease-onset, and a more severe phenotype in males than in females. The phenotype of the *SMPX* c.217dupA carriers was similar to that previously reported in DFNX4 [10]. Males in previous studies of *SMPX* presented with bilateral slowly progressive HL at approximately 2 years of age. However, the affected males from the current family showed hearing impairment at birth or bilateral rapidly progressive late-onset hearing loss, which are still difficult to explain with the vague molecular pathogenic mechanism. The genetic diversity may potentially attribute to stochastic variation, other genetic or environmental factors. As reported, muscle involvement with *SMPX* mutations was also absent in this family [13], and none of the members complained of obvious signs of muscular dysfunction.

SMPX protein has been suggested to be linked to the cytoskeleton and mechanical stress [5]. Thus, *SMPX* is considered to play a protective role against mechanical stress in different cell types of the organ of Corti during the process of hearing [14]. The c.217dupA mutation leads to a frameshift and creates a premature stop codon (PTC) 5 amino acids downstream of the insertion (p.Ile73Asnfs*5). Absence or malfunction of SMPX can result in cumulative damage to cochlea cells, leading to progressive HL, which is consistent with the observed phenotype in this family. The PTC resides 51 nucleotides upstream of the final exon-exon junction, suggesting that the mutated mRNA may be immune to nonsense mediated mRNA decay (NMD) [15]. In that case, the mutated transcripts can continuously produce misfolded mutant proteins and nonfunctional polypeptides, which are harmful to the normal molecular function and lead to HL.

To date, a total of five nonsense mutations of the *SMPX* gene (Fig 2B) have been identified in several cases of X-chromosome-linked NSHL in humans. Moreover, these different mutations were independently identified in families with different racial backgrounds from Newfoundland [16] as well as those with Dutch [13], German and Spanish ancestries [10]. Three of these known mutations were located in exon 3 and two in exon 4. Notably, no mutations has been found in exon 2, and all of these pathogenic variations associated with *SMPX*-related HL were null mutations resulting in different truncated proteins. Surprisingly, the duplication mutation identified in this pedigree was also located in exon 4, and encoded a PTC that causes a truncated protein lacking the C-terminal 11 amino acids. Based on these results, we consider that the *SMPX* gene may be susceptible to this kind of variation, although two potential causative variations were identified in additional sporadic cases of deafness in this study.

*SMPX* was recently suggested to have a founder effect in the Newfoundland population [16]. To investigate its role in the Chinese population, we subsequently screened the coding exons and the flanking sequences of *SMPX* in other unsolved Chinese NSHL pedigrees and sporadic patients. Finally, we identified two missense mutations, including a reported rare variant c.55A>G (p.Asn19Asp) and a de novo variant c.238C>A (p.Leu80Ile), in two sporadic patients with congenital HL unrelated to *GJB2*, *GJB3* and mtDNA mutations. The two residues were highly conserved across species (Fig 2E), and the two substitutions were both deleterious among three functional prediction methods (including SIFT, PolyPhen-2 and MutationTaster). Although c.55A>G has been detected in aggregated populations (ExAC_0.3), it was not found in Chinese normal-hearing controls. The clinical significance of this mutation was previously unknown, and its minor allele frequency (MAF) is low at 0.0001/13 in the ExAC database (MAF is 0.0018/12 in the East Asian population), indicating that this SNP is very rare in the population. Based on our results, we hypothesize that the two substitutions may lead to pathogenicity and therefore the HL phenotype. Moreover, our findings suggest that *SMPX* may play an underestimated role in Chinese deafness.

In conclusion, our study is the first to identify a novel c.217dupA *SMPX* mutation as a monogenetic cause of X-linked NSHL in a Chinese family, which cause not only nonsyndromic late-onset progressive HL, but also congenital hearing impairment. The identification of two *SMPX* variants, a rare variant c.55A>G and a de novo variant c.238C>A, in additional sporadic subjects indicated that this gene should be considered in sporadic patients with inherited congenital hearing impairment. Our results increase the mutation spectrum of the *SMPX* gene, enrich the observed phenotypes of *SMPX*-linked deafness, and may contribute to improve mutation-based genetic counselling for affected families.

## Supporting information

S1 Table Summary of WES data for each sample. (DOCX)

S2 Table Primers for sequencing analysis of the *SMPX* gene. (DOCX)
