# Supplementary material for: A novel frameshift mutation of SMPX causes a rare form of X-linked nonsyndromic hearing loss in a Chinese family
Source: PLoS One. 2017 May 25;12(5):e0178384. doi: 10.1371/journal.pone.0178384 (PMC5444825; doi:10.1371/journal.pone.0178384)
Supplement: S1 Table — (DOCX) [file pone.0178384.s001.docx]

**Table S1** Summary of WES data for each sample

| **Exome Capture Statistics** | **IV: 1**  **(Affected)** | **III: 9**  **(Affected)** | **IV: 3**  **(Affected)** |
| --- | --- | --- | --- |
| Gender | M | M | M |
| Total reads | 43958510 | 42514360 | 43323694 |
| Duplication reads and rate (%) | 3471772 (7.91%) | 4362267 (10.27%) | 4173040 (9.64%) |
| **Reads mapped to genome and rate (%)** | **43903704 (99.88%)** | **42464493 (99.88%)** | **43291229 (99.93%)** |
| With mate mapped to a different chr and rate (%) | 123822 (0.28%) | 142446 (0.34%) | 148410 (0.34%) |
| Initial bases on target (bp) | 50390601 | 50390601 | 50390601 |
| Initial bases near target (bp) | 73902222 | 73902222 | 73902222 |
| Total effective reads | 44019823 | 42584900 | 43414140 |
| Total effective yield (Mb) | 6527.27 | 6317.56 | 6438.90 |
| Effective sequences on target (Mb) | 3801.66 | 3978.20 | 4006.22 |
| Effective sequences near target (Mb) | 1466.61 | 1508.85 | 1602.30 |
| Fraction of effective bases on target (%) | 58.2% | 63.0% | 62.2% |
| Fraction of effective bases on or near target (%) | 80.7% | 86.9% | 87.1% |
| **Average sequencing depth on target (X)** | **75.44** | **78.95** | **79.50** |
| Average sequencing depth near target (X) | 19.85 | 20.42 | 21.68 |
| Mismatch rate in target region (%) | 0.62% | 0.61% | 0.66% |
| Mismatch rate in all effective sequence (%) | 0.53% | 0.51% | 0.55% |
| Base covered on target (bp) | 50333149 | 50333417 | 50331775 |
| **Coverage of target region (%)** | **99.9%** | **99.9%** | **99.9%** |
| Base covered near target (bp) | 70949899 | 71032761 | 71773068 |
| Coverage of flanking region (%) | 96.0% | 96.1% | 97.1% |
| Fraction of target covered >=20X (%) | 95.2% | 96.2% | 96.4% |
| **Fraction of target covered >=10X (%)** | **98.9%** | **99.1%** | **99.1%** |
| Fraction of target covered >=4X (%) | 99.7% | 99.7% | 99.7% |
| Fraction of flanking covered >=20X (%) | 35.3% | 36.4% | 38.8% |
| Fraction of flanking covered >=10X (%) | 56.9% | 57.7% | 60.9% |
| Fraction of flanking covered >=4X (%) | 80.5% | 80.7% | 83.7% |
