# Supplementary material for: A novel frameshift mutation of SMPX causes a rare form of X-linked nonsyndromic hearing loss in a Chinese family
Source: PLoS One. 2017 May 25;12(5):e0178384. doi: 10.1371/journal.pone.0178384 (PMC5444825; doi:10.1371/journal.pone.0178384)
Supplement: S2 Table — (DOCX) [file pone.0178384.s002.docx]

**Table S2** Primers for sequencing analysis of the *SMPX* gene

| Exons | Primer (5’ to 3’) |
| --- | --- |
| Exon -2F | CGAGGCGTCTCACCATTTC |
| Exon -2R | GCAGGGCTACTTACCTGGATG |
| Exon -3F | GCTTTGAAGATGTACTTTTCAGACG |
| Exon -3R | AAGGGAAGGGAGAAGGCAGT |
| Exon -4F | GAAACTCTAAGGCTGTCTCAACA |
| Exon -4R | GGAAGGCTTCCTTAAACCATTT |

F, forward primer; R, reverse primer
